# Management of Alcohol and Tobacco Use Disorders in a 39-Year-Old Hispanic Male With a Complex Medical Background: A Case Report

**DOI:** 10.7759/cureus.60930

**Published:** 2024-05-23

**Authors:** Farhana Nazmin, Jayanta Chowdhury

**Affiliations:** 1 Psychiatry, BronxCare Health System (BCHS), Bronx, USA

**Keywords:** psychiatric comorbidities, tobacco use disorder, alcohol use disorder, bariatric surgery, substance use disorder

## Abstract

Substance use disorders affect the mental activities of an individual’s brain and behavior, leading to a loss of control over their substance use, such as drugs, alcohol, and medication. However, these disorders are treatable. This case report presents and discusses the management of a 39-year-old Hispanic male with a complex medical background and a history of substance use. The patient, who resided with his mother in the Bronx, was admitted to the Outpatient Program (OPD) at the Life Recovery Center (LRC) Addiction Treatment Center for concurrent alcohol and tobacco use disorders. The patient had a history of anemia after bariatric surgery 10 years ago and no significant psychiatric history. Therefore, a comprehensive approach was required for the patient's treatment. The case further highlights the patient's presentation, treatment options, medication, and outcomes, which are essential for managing substance use disorders in individuals with complex medical backgrounds.

## Introduction

Continuous alcohol and tobacco use disorders present a significant challenge in addiction treatment, particularly in individuals with more than one medical condition [1]. Bariatric surgery, which is conducted for weight loss, can further complicate the treatment of substance use disorders due to modifications in metabolism and absorption [2]. Substance use disorders (SUDs) are chronic, relapsing conditions characterized by addictive and excessive behaviors despite harmful consequences [1]. Substances, including different types of alcohol, tobacco, opioids, and stimulants, pose negative impacts on physical, psychological, and social well-being [3]. SUDs can be diagnosed using criteria mentioned in the Diagnostic and Statistical Manual of Mental Disorders (DSM-5), which includes impaired control, social impairment, risky use, and pharmacological criteria [1].

Tobacco use disorders are chronic, relapsing conditions that not only have a severe impact on physical health but also alter psychological and social well-being. Following bariatric surgery, the changes in the body's handling of substances can affect how tobacco products are metabolized. This alteration might influence nicotine cravings and the efficacy of pharmacological treatments designed to manage tobacco dependence [1]. The disorders occur through continuous use and withdrawal symptoms after concluding substance use, with unsuccessful attempts to quit or reduce substance use despite the negative consequences [4]. SUDs can also lead to psychiatric comorbidities such as anxiety, depression, and personality disorders, which further complicate treatment and comorbid medical conditions [5]. The treatment of SUDs can be achieved by administering drugs, behavioral interventions, and support services. Pharmacology plays a crucial role in the management of SUDs by targeting neurobiological mechanisms underlying medical conditions caused by addiction [6]. The management of SUDs through medications is available for various substances, including nicotine, alcohol, and opioids. Approved for the treatment of alcohol use disorder, medications like naltrexone, acamprosate, and disulfiram are known to reduce cravings and lower relapse rates [7]. Similarly, drugs such as methadone, buprenorphine, and naltrexone are utilized in opioid use disorder treatment, alleviating withdrawal symptoms and cravings while supporting long-term recovery [8]. Hence, behavioral interventions are fundamental components in SUD treatments, used to modify maladaptive behaviors, enhance coping skills, and promote sustained abstinence [9].

Post-bariatric surgery substance use disorder

Bariatric surgery, which includes procedures such as gastric bypass and sleeve gastrectomy, is often performed to achieve weight loss in obese individuals [10]. These surgeries modify the structure and physiology of the gastrointestinal tract, allowing the individual to consume less food and alter nutrient absorption and the hormone regulation system. Bariatric surgery has the advantage of promoting weight loss and improving obesity-related conditions; however, it can also have unintended consequences, such as modifying alcohol metabolism and absorption [11]. Post-bariatric surgery SUD refers to the development or expansion of substance use behaviors following bariatric surgery [12]. Several factors may contribute to the increased risk of SUDs in people, including modified neurobiological responses to substances, changes in reward pathways, and psychological factors, such as stress and anxiety [13]. Additionally, changes in alcohol pharmacokinetics post-surgery, including increased peak blood alcohol concentrations and prolonged intoxication, may contribute to the development of an alcohol use disorder [14].

## Case presentation

A 39-year-old single Hispanic male who domiciled with his mother in a private residence in the Bronx has a history of alcohol and tobacco use disorders as well as anemia and underwent bariatric surgery 10 years ago. The patient has no substantial psychiatric history. The patient was referred from the Life Recovery Center (LRC) Inpatient Detox Unit to the LRC Addiction Outpatient Program, where he was admitted to the OPD on October 30, 2023. The laboratory findings and pathology report of the patient are shown in Table 1.

**Table 1:** Laboratory results of the patient characteristics AST: Aspartate transaminase; ALT: Alanine transaminase.

Parameters	Result
WBC count	6.9 k/μL
RBC count	4.88 MIL/μL
Hemoglobin (HGB)	9.9 g/dL
Hematocrit	32.6%
Mean corpuscular volume (MCV)	66.7 fL
Mean corpuscular hemoglobin (MCH)	20.2 pg
Mean corpuscular hemoglobin concentration (MCHC)	30.3 g/dL
Mean platelet volume (MPV)	7.4 fL
Red cell distribution width (RDW)	19.8%
Platelet count	307 k/μL
Neutrophil percentage (neutro%)	68.2%
Neutrophil count (neutro count)	4.7 k/μL
Lymphocyte percentage (lymphocyte%)	22.4%
Lymphocyte count	1.5 k/μL
Monocyte percentage (monocyte%)	7.9%
Monocyte count	0.5 k/μL
Eosinophil percentage (eosinophil%)	1.1%
Eosinophil count	0.10 k/μL
Basophil count	0.0 k/μL
Calcium	9.5 mg/dL
Total protein (serum)	7.7 g/dL
Albumin (serum)	4.3 g/dL
Aspartate transaminase (AST, serum)	23 U/L
Alkaline phosphatase (serum)	68 U/L
Total bilirubin (serum)	0.3 mg/dL
Direct bilirubin (serum)	<0.2 mg/dL
Alanine aminotransferase (ALT, serum)	22 U/L
Unsaturated iron binding capacity (UIBC)	543 μg/dL
Iron (serum)	18 μg/dL
Vitamin D (25 OH, total)	21.6 ng/mL
Serum ferritin	6.8 ng/mL
Vitamin B12 (serum)	407 pg/mL
Folate (serum)	7.1 ng/mL

Diagnosis

The patient has been diagnosed with multiple conditions such as moderate alcohol use disorder (ICD-10 CM F10.20), tobacco use disorder (ICD-10 CM F17.210), status post-gastric bypass for obesity (ICD-10-CM Diagnosis Code K91.1), and iron deficiency anemia (ICD-10-CM D50.9).

Abnormal presence

The patient exhibited abnormal laboratory results, including low levels of hemoglobin (HGB), hematocrit, mean corpuscular volume (MCV), vitamin D, serum ferritin, and folate (Table 2).

**Table 2:** Abnormal findings AST: Aspartate transaminase; ALT: Alanine transaminase.

Diagnostic finding	Interpretation
Low hemoglobin	Potential indication of anemia, possibly related to chronic substance abuse
Low hematocrit	Often accompanies low hemoglobin, suggestive of anemia
Low mean corpuscular volume (MCV)	Indicates smaller than normal red blood cells, possibly due to chronic substance abuse
Low vitamin D	Indicates vitamin D deficiency, which can be exacerbated by substance abuse
Low serum ferritin	Suggests iron deficiency, a common consequence of chronic substance abuse
Low folate	Indicates folate deficiency, which can result from poor nutrition associated with substance abuse
Elevated liver enzymes (AST, ALT)	Suggestive of liver dysfunction, potentially caused by alcohol abuse or drug-induced hepatotoxicity

Substance use information

The patient reported that he first drank alcohol at the age of seven, claiming that he was “forced.” During his 20s, he began consuming hard liquor, particularly during national holidays. A notable turning point occurred after bariatric surgery a decade ago when his daily alcohol intake escalated, initially after indulging in a couple of beers with friends. A previous study has shown that alcohol intake and substance use increased in individuals after bariatric surgeries [15].

Subsequently, our patient had a progressively worsening history of alcohol consumption over the following eight years after surgery. The patient acknowledges a significant “drinking problem,” revealing that he consumed seven liters of whiskey weekly in addition to a few beers daily. His most recent drink occurred before seeking assistance at the BronxCare Hospital. Following a referral from the Jacobi Hospital, he was directed to BronxCare for inpatient detoxification. He also reports occasional blackouts after heavy drinking. The patient currently smokes, consuming approximately one packet of cigarettes per day. He denies intravenous drug use or using recreational substances.

The patient has no prior history of seeking treatment or detoxification services for alcohol use except for the current inpatient detoxification administration. Notably, the patient has reportedly maintained sobriety for one year, from 2015 to 2016, which he claims was facilitated by avoiding serious relationships and refraining from socializing, while the only desire to drink was to prevent withdrawal symptoms. However, he did engage with a counselor at Montefiore earlier in 2023 for substance use disorder but had to discontinue due to work commitments. He actively participated in groups and Alcoholics Anonymous (AA) meetings and attended evening groups at LRC.

Chief complaint

The patient presented with the chief complaint, "I need help; I have a drinking problem."

Psychiatric history

The patient refused any prior inpatient psychiatric admissions, visits to Comprehensive Psychiatric Emergency Programs (CPEP), or any follow-up without a patient psychiatrist/therapist. There was no history of using other psychotropic medications. The patient denied experiencing hallucinations or delusions.

In 2020, the patient suffered a panic attack in his office upon realizing he was the sole occupant. Mistakenly perceiving symptoms of a heart attack, emergency services were summoned, leading to an emergency room (ER) visit. He was discharged after two hours without receiving any medication. Panic disorders are primarily associated with SUDs, with an odd ratio of 1.7 to 4.1 [16]. The patient shared the distressing information that a friend died by suicide in 2016, with whom he had spent time on the same night as the incident; this event has created feelings of guilt.

During his upbringing, the patient endured physical abuse from his mother's boyfriend. However, he denied exhibiting symptoms of post-traumatic stress disorder, such as hypervigilance, being easily startled, flashbacks, or nightmares. The patient denied symptoms of panic attacks, social anxiety, specific anxiety, major depressive disorder (MDD), or mania/hypomania. There were no reported prior suicide attempts or self-injury behaviors. Furthermore, the patient denied any historical or present issues with gambling.

While expressing overall well-being, the patient acknowledged job-related stressors, stating, "I have a stressful job; lots of people rely on me."

Medical history

The patient has no known allergies. The patient disclosed a history of bariatric surgery at the age of 29, when his weight had reached 400 pounds. After the surgery, he successfully lost 155 pounds and currently weighs 350 pounds with a BMI of 46. However, post-surgery, his alcohol intake notably increased, progressing from casual beer consumption to daily intake of hard liquor. Studies have indicated a potential increase in substance use following bariatric surgery due to changes in the body's ability to metabolize alcohol, often leading to quicker intoxication and a greater reinforcement of alcohol use [17]. A recent left ankle sprain was reported, along with concerns about a potential blood clot in his leg. His primary care physician conducted the evaluation and management. The patient's last follow-up occurred in March 2023. Additionally, the patient was under the supervision of a vascular surgeon, who, after a prior ultrasound, did not recommend any specific interventions or treatments.

Surgical history

The patient underwent billable/specific bariatric surgery 10 years ago. The patient was recommended to follow up with his dietician.

Family history

The patient denied any family history of mental illness or suicidal attempts but acknowledged that his oldest sister had an opioid use disorder. However, the patient currently has no contact with her. The patient underscored that they receive substantial support from their family, which motivates them to seek assistance. Additionally, the patient recognized the vital role played by a network of friends who provide valuable help and support. The patient experienced a significant impact due to family circumstances, undertaking numerous responsibilities during their childhood following their father's death. The dynamics within the family continued to affect the patient profoundly. The patient denied having any legal case against him.

Psychosocial history

The patient was born and raised in the Bronx, New York. He was single with no children. He was residing with his mother, having maintained the same living arrangement for the past two decades. The patient completed college and attained a master's degree. He denied participation in special classes or any history of vocational training. He supports himself financially through government employment. His prognosis is guarded but favorable.

Mental status examination

The patient is a Hispanic male, well-groomed, appearing his stated age, weighing 350 pounds (overweight), and appropriately dressed. He was fully conscious and fully oriented, ambulating independently. During the interview, he was cooperative and made appropriate eye contact. The patient had an appropriate affect and did not appear internally preoccupied. The speech was clear with normal rate and volume. No delusion was elicited. He currently denied suicidal/homicidal ideation. His intelligence and cognition were average. Memory and abstract thinking were within normal limits. He presented with fair-good insight and judgment.

## Discussion

Medical history and clinical course

After discharge from the LRC Detoxification Center on September 24, 2023, the patient was sober for two weeks but admitted to having persistent urges to drink thereafter. His last episode involved one liter of whiskey and four beers over the last weekend in October 2023. However, he reported that he did not experience any pleasure from drinking but suffered from nausea, headache, and lightheadedness after drinking. Additionally, he noted that he sometimes drank to avoid withdrawal symptoms and also got anxious about missing work days due to his alcohol consumption. The patient also reported difficulty falling and maintaining sleep, sometimes waking up in the middle of the night with shortness of breath. He was encouraged to follow up with his primary care physician (PCP) for obstructive sleep apnea (OSA) evaluation. The review of the system and physical examination findings revealed unremarkable thyroid disorder, infectious disease, or any malignancy. According to the laboratory report, he has iron and vitamin D deficiencies and anemia.

The patient was educated about potential lifestyle changes; obesity is a risk factor for metabolic syndrome. Additionally, the patient was informed of nutritional deficiencies from chronic alcoholism following bariatric surgery.

The patient was interested in starting medications for his alcohol use disorder. We discussed different medications, including their potential side effects. Subsequently, the patient was prescribed naltrexone 50 mg oral tablet, folic acid 1 mg oral tablet, thiamine 100 mg oral tablet, multiple oral vitamin tablets, and ferrous sulfate 325 mg (65 mg elemental iron) oral tablet.

The combination of increased alcohol consumption and smoking post-bariatric surgery has had detrimental effects on the patient's weight management. Alcohol contains empty calories, which can contribute to weight regain, and smoking has been shown to alter metabolism, potentially complicating the maintenance of weight loss post-surgery [18]. His current weight was 350 pounds, with a BMI of 46, indicating that he has regained some of the weight lost post-surgery.

The increase in alcohol consumption and continued smoking after bariatric surgery significantly impacted the patient's weight management. Alcohol, rich in empty calories, contributes to weight regain, complicating post-surgery weight management [19]. Additionally, smoking has been linked to metabolic disturbances that can affect weight. Nicotine increases the metabolic rate, which might initially lead to weight loss but can disrupt long-term weight management strategies post-bariatric surgery by increasing appetite and caloric intake once smoking is ceased [20].

## Conclusions

This case report demonstrates the effectiveness of personalized treatment, combining medication and therapy, in helping people with complex medical histories, such as those who have undergone bariatric surgery, to overcome SUDs. The improvements in their health and happiness highlight the power of tailored care and support. This experience underlines the need for further research to refine these methods and help more people in the future.

## References

[1] American Psychiatric Association (2013). Diagnostic and Statistical Manual of Mental Disorders, Fifth Edition.

[2] Courcoulas AP, Christian NJ, Belle SH (2013). Weight change and health outcomes at 3 years after bariatric surgery among individuals with severe obesity. JAMA.

[3] (2023). Drugs, brains, and behavior: the science of addiction. https://nida.nih.gov/research-topics/addiction-science/drugs-brain-behavior-science-of-addiction.

[4] National Institute on Drug Abuse (2018). Principles of Drug Addiction Treatment: A Research-Based Guide (Third Edition). https://nida.nih.gov/sites/default/files/podat-3rdEd-508.pdf.

[5] Swendsen J, Conway KP, Degenhardt L (2010). Mental disorders as risk factors for substance use, abuse and dependence: results from the 10-year follow-up of the National Comorbidity Survey. Addiction.

[6] Volkow ND, Morales M (2015). The brain on drugs: from reward to addiction. Cell.

[7] Jonas DE, Amick HR, Feltner C (2014). Pharmacotherapy for adults with alcohol use disorders in outpatient settings: a systematic review and meta-analysis. JAMA.

[8] Mattick RP, Breen C, Kimber J, Davoli M (2003). Methadone maintenance therapy versus no opioid replacement therapy for opioid dependence. Cochrane Database Syst Rev.

[9] McHugh RK, Hearon BA, Otto MW (2010). Cognitive behavioral therapy for substance use disorders. Psychiatr Clin North Am.

[10] Colquitt JL, Pickett K, Loveman E, Frampton GK (2014). Surgery for weight loss in adults. Cochrane Database Syst Rev.

[11] King WC, Chen JY, Mitchell JE (2012). Prevalence of alcohol use disorders before and after bariatric surgery. JAMA.

[12] Woodard GA, Downey J, Hernandez-Boussard T, Morton JM (2011). Impaired alcohol metabolism after gastric bypass surgery: a case-crossover trial. J Am Coll Surg.

[13] King WC, Chen JY, Courcoulas AP (2017). Alcohol and other substance use after bariatric surgery: prospective evidence from a U.S. multicenter cohort study. Surg Obes Relat Dis.

[14] Wadden TA, Foster GD (2000). Behavioral treatment of obesity. Med Clin North Am.

[15] Engel SG, Schaefer LM, Kerver GA (2022). The rewarding effects of alcohol after bariatric surgery: do they change and are they associated with pharmacokinetic changes?. Surg Obes Relat Dis.

[16] Smith JP, Randall CL (2012). Anxiety and alcohol use disorders: comorbidity and treatment considerations. Alcohol Res.

[17] Azam H, Shahrestani S, Phan K (2018). Alcohol use disorders before and after bariatric surgery: a systematic review and meta-analysis. Ann Transl Med.

[18] Chow A, Neville A, Kolozsvari N (2021). Smoking in bariatric surgery: a systematic review. Surg Endosc.

[19] Samaan JS, Mohan S, Toubat O (2021). Effects of smoking on bariatric surgery postoperative weight loss and patient satisfaction. Surg Endosc.

[20] Khodamoradi F, Nazemipour M, Mansournia N, Yazdani K, Khalili D, Mansournia MA (2021). The effects of smoking on metabolic syndrome and its components using causal methods in the Iranian population. Int J Prev Med.

